# Microwave-Assisted Extraction of Rubusoside from *Rubus chingii* var. *suavissimus* Leaves Using a Recyclable Ternary Deep Eutectic Solvent: Process Optimization and Mechanistic Insights

**DOI:** 10.3390/foods15142545

**Published:** 2026-07-19

**Authors:** Heyao Liang, Zhenjiang Jin, Chengxi Yang, Ziyuan Li, Weijian Chen, Wu Yuan

**Affiliations:** 1 Guangxi Key Laboratory of Environmental Pollution Control Theory and Technology, Guilin University of Technology, Guilin 541006, China; 17677135448@163.com (H.L.); 18272131709@163.com (C.Y.); jj327_2005@163.com (W.C.); wuyuan194@163.com (W.Y.); 2 University Engineering Research Center of Watershed Protection and Green Development, Guilin University of Technology, Guilin 541006, China; 3 Key Laboratory of Carbon Emission and Pollutant Collaborative Control, Education Department of Guangxi Zhuang Autonomous Region, Guilin University of Technology, Guilin 541006, China; 4 College of Chemistry and Bioengineering, Guilin University of Technology, Guilin 541004, China; 2001028@glut.edu.cn

**Keywords:** *Rubus chingii* var. *suavissimus*, rubusoside, ternary deep eutectic solvent, microwave-assisted extraction, DFT, recovery

## Abstract

Rubusoside is the major sweet bioactive compound in *Rubus chingii* var. *suavissimus* (S.K.Lee) L.T.Lu, characterized by high sweetness, low caloric value, and favorable safety, with potential applications as a natural sweeteners and in functional foods. However, efficient green extraction technologies and their mechanisms remain insufficiently explored. Here, a microwave-assisted deep eutectic solvent (DES) system was developed for rubusoside recovery. The ternary DES composed of choline chloride, 1,2-propylene glycol, and 1,3-butanediol (1:2:2) showed the best performance and outperformed microwave-assisted water extraction. Response surface methodology identified optimal conditions of 33% moisture content, a liquid–solid ratio of 21 mL/g, 6 min, and 320 W, yielding 7.89 ± 0.25% rubusoside. Fourier-transform infrared spectroscopy, electrostatic potential, atoms-in-molecules theory, and independent gradient modelling based on Hirshfeld partition analyses revealed significant non-covalent interactions between the ternary DES and rubusoside. Scanning electron microscopy showed that DES and microwave treatment synergistically disrupted plant tissues and enhanced mass transfer. LX-28 macroporous resin enabled rubusoside separation, and the recovered DES retained stable performance after five reuse cycles. These results demonstrate a green, efficient, and recyclable strategy driven by cooperative hydrogen bonding and van der Waals interactions between the ternary DES and the rubusoside glycosyl moiety, together with DES–microwave-induced tissue disruption and mass-transfer enhancement.

## 1. Introduction

*Rubus chingii* var. *suavissimus* (S.K.Lee) L.T.Lu (Guangxi sweet tea) is a perennial deciduous shrub belonging to the genus Rubus in the family Rosaceae [1]. It is naturally distributed mainly on hillsides in Guangxi Zhuang Autonomous Region, southern China, and has long been used as a traditional medicinal tea plant in this region. The leaves of *Rubus chingii* var. *suavissimus* are rich in rubusoside, a diterpene glycoside with the molecular formula C_32_H_50_O_13_ [2]. As the major sweet component of *R. chingii* var. *suavissimus*, rubusoside exhibits high sweetness, low caloric value and favorable nutritional characteristics, showing great potential as a natural green alternative to conventional sweeteners such as sucrose and saccharin. With the increasing global demand for natural, nutritious and multifunctional foods, the development and application of novel sweeteners have attracted growing attention [3]. Therefore, developing efficient and green extraction technologies for rubusoside is of great significance for promoting the high-value utilization of Guangxi sweet tea resources and the development of related products.

At present, rubusoside is mainly obtained from *R. chingii* var. *suavissimus* through water extraction, alcohol extraction and subsequent separation and purification processes. However, traditional extraction methods generally suffer from low extraction efficiency, high solvent consumption and considerable environmental burden [4]. Microwave-assisted extraction (MAE), as a green extraction technology, enables rapid energy transfer and simultaneous heating of biological materials and solvent systems [5]. Compared with conventional extraction methods, MAE shortens extraction time, reduces solvent consumption and improves extraction yield [6,7]. In recent years, MAE has been successfully applied to the efficient recovery of and yield improvement in various bioactive compounds and natural products, including polyphenols, saponins and pectin [8,9,10]. Nevertheless, the extraction efficiency of MAE strongly depends on the solvent system. Conventional organic solvents are often associated with high volatility, toxicity and poor environmental compatibility, which limits the full realization of the green advantages of MAE. In this context, DESs have received increasing attention as a new class of green alternative solvents [11].

DESs are homogeneous liquid mixtures generally prepared by combining hydrogen bond acceptors (HBAs) and hydrogen bond donors (HBDs) at appropriate molar ratios under constant-temperature stirring and heating conditions [12]. In addition to their green characteristics, such as low volatility, low toxicity, biodegradability and antibacterial properties [13,14], DESs also possess excellent thermochemical stability, strong solvation ability and high selectivity, and have been widely used in organic synthesis, electrochemistry, catalysis, separation and extraction [15]. Previous studies have shown that DESs can efficiently extract flavonoids and phenolic bioactive compounds from plants [16] and have also been widely applied in the extraction and separation of terpenoids and saponins [17,18]. In addition, Tang [19] used DESs to extract tea saponins from *Camellia oleifera* seed pomace, achieving significantly better performance than traditional solvent extraction. Meanwhile, characterization methods such as Fourier transform infrared spectroscopy (FTIR), scanning electron microscopy (SEM), density functional theory (DFT)-based electrostatic potential (ESP) analysis, atoms-in-molecules (AIM) theory and the independent gradient model based on Hirshfeld partition (IGMH) have gradually been applied to DES systems. These approaches help reveal hydrogen bonding, van der Waals interactions and electron density redistribution between DESs and target compounds, thereby elucidating the mechanisms by which DESs enhance dissolution and extraction at the molecular level. However, studies on the microwave-assisted DES extraction of diterpene glycosides remain limited, and the interaction behavior between DESs and rubusoside, as well as the corresponding extraction enhancement mechanism, has not yet been systematically investigated. Rubusoside, the major sweet bioactive compound in Guangxi sweet tea, is a typical diterpene glycoside containing both a hydrophobic diterpene skeleton and a hydroxyl-rich glycosyl moiety, which gives it an amphiphilic molecular structure [20]. The abundant hydroxyl groups in its glycosyl moiety endow rubusoside with strong polarity and hydrogen bonding ability, enabling extensive intermolecular interactions with polar solvents [21]. In addition, polar molecules and ionic systems can efficiently absorb microwave energy and generate rapid dielectric heating [22]. Therefore, based on its structural characteristics, the abundant hydrogen-bond donor and acceptor sites in DESs are expected to enhance the solubility of rubusoside, while microwave-enhanced mass transfer may further promote its release.

Based on the above considerations, we hypothesized that the synergistic effect between DESs and microwave treatment could improve the dissolution, diffusion and release of rubusoside, thereby enabling its efficient and green extraction. To verify this hypothesis, the present study was designed as follows: (1) ChCl was used as the HBA, and different types of HBDs were screened to construct DES systems suitable for rubusoside extraction, followed by comparison with microwave-assisted water extraction; (2) FTIR was used to characterize the optimal DES and different extraction products, and DFT calculations were combined to analyze the interaction mechanism between DESs and rubusoside at the molecular level; (3) single-factor experiments and response surface methodology (RSM) were further applied to optimize key extraction parameters, while SEM was used to observe the microstructural changes of raw materials and extraction residues to reveal the synergistic extraction enhancement mechanism of DES and microwave treatment; and (4) the separation, recovery and recyclability of DES were evaluated. Therefore, this study aimed to construct a microwave-assisted DES extraction system, systematically evaluate its extraction performance for rubusoside from *R. chingii* var. *suavissimus*, clarify the underlying mechanism and assess the recyclability of DES, thereby providing a theoretical basis for the design and development of green extraction systems for glycoside-type natural products.

## 2. Materials and Methods

### 2.1. Raw Materials and Chemicals

Commercially available dried leaves of Guangxi sweet tea (*R. chingii* var. *suavissimus*) were purchased from Guangxi Jinxiu Xingrong Tea Co., Ltd. (Laibin, China) in 2024. The leaves were further dried at 60 °C for 24 h, ground into powder, passed through a 40-mesh sieve, and stored in sealed bags until further use. Choline chloride (ChCl), acetic acid, citric acid, D-sorbitol, glycerol, ethylene glycol, levulinic acid, lactic acid, malic acid, diethylene glycol, 1,2-propylene glycol (1,2-PG), and 1,3-butanediol (1,3-BD) were purchased from Shanghai Macklin Biochemical Technology Co., Ltd. (Shanghai, China). Urea and glucose were purchased from Xilong Scientific Co., Ltd. (Shantou, China). Methanol (MeOH) and acetonitrile (ACN) were purchased from Anhui Tiandi Life Technology Co., Ltd. (Anqing, China). Macroporous adsorption resins and the rubusoside reference standard (purity ≥ 98%, HPLC) were purchased from Shanghai Yuanye Bio-Technology Co., Ltd. (Shanghai, China). Unless otherwise specified, all reagents were of analytical grade.

### 2.2. Synthesis of DESs

All DESs used in this study were prepared by mixing ChCl as the hydrogen bond acceptor (HBA) with different types of hydrogen bond donors (HBDs) at predetermined molar ratios. The components were accurately weighed and transferred into sample vials containing magnetic stir bars. The mixtures were then heated and stirred at 80 °C for at least 3 h using a thermostatic magnetic stirrer until clear and homogeneous liquid systems were formed [23,24]. The prepared DESs were transferred into sealed glass bottles and stored in an oven at 80 °C until further use. Before use, ultrapure water was added at a DES-to-water mass ratio of 3:1, and the mixture was continuously stirred until a completely transparent system was obtained. The density of the DES was measured using a density meter. The viscosity of the DES at different temperatures was determined using a BROOKFIELD DVNXHBCBG rotational viscometer equipped with spindle No. 40 at a rotation speed of 250 r/min.

### 2.3. Optimization of DES Extraction Process

**Figure 1:**
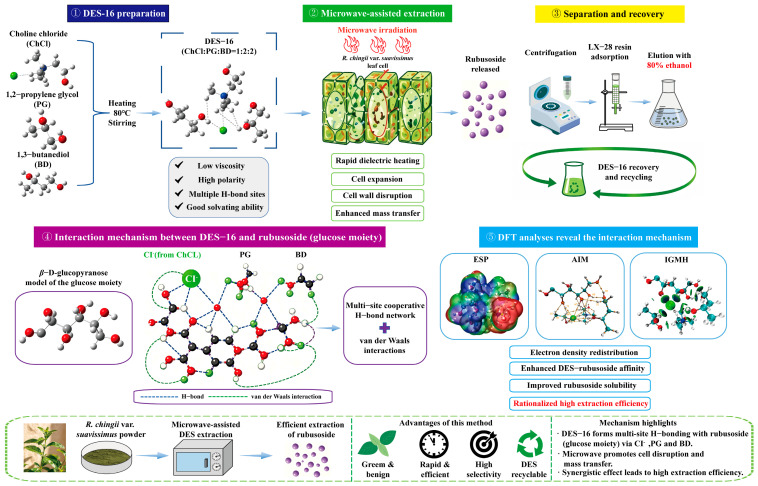
Schematic Representation of Microwave-Assisted DES-16 Extraction of Rubusoside and Its Underlying Molecular Mechanism.

The overall experimental workflow for microwave-assisted DES extraction, rubusoside separation, DES recovery, and mechanistic analysis is illustrated in Figure 1. The prepared DES was thoroughly mixed with *R. chingii* var. *suavissimus* powder that had passed through a 40-mesh sieve, and the mixture was then subjected to microwave heating using a microwave oven (Midea, MM8MEFQ3-PR). Microwave power and irradiation time were varied according to the single-factor experiments and Box–Behnken design. During verification under the optimized conditions, extraction was conducted at 320 W for 6 min. The extraction temperature was not independently fixed because the microwave system was operated in power-controlled mode without external temperature control. Previous studies have shown that the extraction efficiency of rubusoside using DES is affected by various process variables, among which liquid–solid ratio, DES moisture content, extraction time and microwave power are key factors influencing extraction performance [25]. Therefore, single-factor experiments were conducted in this study to investigate the effects of these parameters. Briefly, 1 g of *R. chingii* var. *suavissimus* powder was thoroughly mixed with the DES that showed the highest rubusoside extraction yield, while the other extraction parameters were kept constant. Then, only one variable was adjusted at a time to identify the main factors that significantly affected rubusoside extraction yield. Based on the single-factor experiments, DES moisture content, liquid–solid ratio, microwave time, and microwave power were selected as the independent variables, and rubusoside extraction yield was used as the response. A four-factor, three-level Box–Behnken design was employed for response surface optimization. The coded and actual levels of the independent variables are presented in Table A2. A second-order polynomial model was fitted to the experimental data, and numerical optimization was performed to maximize rubusoside extraction yield. The predicted optimal conditions were subsequently verified experimentally. Experimental design, regression analysis, analysis of variance, and numerical optimization were performed using Design-Expert 13 software (Stat-Ease Inc., Minneapolis, MN, USA).

### 2.4. Rubusoside Separation and Recovery of DES for Recycling

The rubusoside separation and DES recovery procedures are illustrated in Step 3 of Figure 1. After microwave-assisted extraction, the extract was centrifuged at 4000 r/min for 10 min, and the supernatant was loaded onto an LX-28 macroporous resin column at 0.5 mL/min. Rubusoside was adsorbed onto the resin, while the DES-containing effluent was collected for recovery. After washing with deionized water, rubusoside was eluted with 80% ethanol at 0.5 mL/min. The recovered DES was concentrated, adjusted to the required moisture content, and reused in subsequent extraction cycles.

### 2.5. Quantitative Determination and Purity Analysis of Rubusoside by HPLC

Rubusoside was quantitatively determined using an Agilent 1260 high-performance liquid chromatography (HPLC) system (Agilent Technologies, Inc., Santa Clara, CA, USA). Chromatographic separation was performed on a ZORBAX SB-C18 column (4.6 × 250 mm, 5 μm; Agilent Technologies, Inc., Santa Clara, CA, USA) at a column temperature of 30 °C. Acetonitrile–water (33:67, *v*/*v*) was used as the mobile phase at a flow rate of 1.0 mL/min. The detection wavelength was set at 205 nm, and the injection volume was 10 μL. A total of 5 mg of rubusoside standard was accurately weighed and dissolved in ultrapure water to prepare a stock solution with a concentration of 1 mg/mL, which was stored at 4 °C until use. Appropriate volumes of the stock solution were diluted with ultrapure water to obtain standard working solutions at concentrations of 0.02, 0.04, 0.06, 0.08 and 0.10 mg/mL, which were analyzed under the chromatographic conditions described above. A standard calibration curve was established by plotting rubusoside concentration (X) against peak area (Y), yielding the regression equation y = 4014x − 7.2 with a correlation coefficient of R^2^ = 0.9997. For sample analysis, 1 mL of crude rubusoside extract was diluted 25-fold and analyzed under the same HPLC conditions. The rubusoside concentration in the sample solution was calculated according to the calibration curve, and the extraction yield of rubusoside (EYR) was calculated using Equation (1).

(1) $$\mathrm{EYR}\;(\%)=\frac{C\times V\times N}{M\times 1000}\times 100$$

where *C* is the mass concentration of rubusoside (mg/mL), *V* is the volume of the sample solution (mL), *N* is the dilution factor, and *M* is the mass of *R. chingii* var. *suavissimus* powder used for extraction (g).

For purity determination, the rubusoside-enriched fraction obtained after LX-28 resin purification and elution with 80% ethanol was concentrated and freeze-dried. A total of 5.0 mg of the freeze-dried fraction was accurately weighed, dissolved in the mobile phase, and diluted to 10 mL. The resulting solution was further diluted 10-fold with the mobile phase and filtered through a 0.45 μm membrane before HPLC analysis. Rubusoside was identified by comparison of its retention time with that of the reference standard and quantified using the established calibration curve. The rubusoside purity was calculated using Equation (2).

(2) $$\mathrm{Rubusoside}\;\mathrm{purity}\;(\%)\;=\frac{C_{p}\times V_{p}\times N_{p}}{m_{p}}\times 100$$

where *C_p_* is the rubusoside concentration in the analyzed sample solution (mg/mL), *V_p_* is the initial volume of the purified sample solution (mL), *N_p_* is the dilution factor, and *m_p_* is the mass of the freeze-dried purified fraction (mg).

### 2.6. Characterization

#### 2.6.1. Fourier Transform Infrared Spectroscopy Analysis

Fourier transform infrared spectroscopy (FTIR) analysis was performed using an FTIR spectrometer (PerkinElmer, Inc., Waltham, MA, USA). The samples were analyzed using the KBr pellet method over the wavenumber range of 4000–400 cm^−1^, with a resolution of 4 cm^−1^ and 32 scans. The FTIR results were used to analyze changes in functional groups and intermolecular interactions in the DES and extraction products.

#### 2.6.2. Scanning Electron Microscopy Analysis

The microstructures of the raw and extracted *R. chingii* var. *suavissimus* powders were observed using a scanning electron microscope (SEM, SU5000, Hitachi, Japan). Before observation, the samples were sputter-coated with gold under vacuum, and the accelerating voltage was set at 5 kV. The SEM results were used to evaluate the effects of different extraction methods on the plant tissue structure.

### 2.7. Density Functional Theory (DFT) Calculations

To investigate the interaction characteristics between DESs and the glycosyl moiety of rubusoside, the hydroxyl-rich glucose unit in rubusoside was selected as a representative model molecule to construct complex structures with different DESs. All structures were subjected to density functional theory (DFT) calculations using Gaussian 16. Geometry optimization and frequency analysis were performed at the B3LYP-D3(BJ)/6-311+G(d,p) level of theory to ensure that the optimized structures were stable configurations without imaginary frequencies. Subsequently, electrostatic potential (ESP), atoms-in-molecules (AIM) theory and independent gradient modelling based on Hirshfeld partition (IGMH) analyses were performed on the optimized structures using Multiwfn 3.8 [26]. Specifically, ESP analysis was used to characterize the surface charge distribution and potential interaction sites of the systems; AIM theory [27] was employed to calculate the properties of bond critical points (BCPs) between DESs and the glucose model; and IGMH analysis was used to visualize the spatial distribution of non-covalent interactions. Combined with sign(λ_2_)ρ scatter plots, IGMH further enabled the analysis of hydrogen bonding, van der Waals interactions and steric effects. All molecular structures and isosurface maps were visualized using VMD 1.9.4 [28].

### 2.8. Statistical Analysis

All experiments were performed in triplicate, and the results are expressed as mean ± standard deviation. Analysis of variance (ANOVA) and Duncan’s multiple range test were conducted using SPSS Statistics 27.0.1, with *p* < 0.05 considered statistically significant. Figures and tables were prepared and organized using Origin 2024, Adobe Illustrator (version 28.7.1, Adobe Inc., San Jose, CA, USA) and Microsoft Excel 2021.

## 3. Results

### 3.1. Screening of DES

To ensure comparability, all candidate DESs were screened at predefined molar ratios under identical extraction conditions, and the best-performing formulation was subsequently subjected to molar-ratio optimization. ChCl was used as the hydrogen bond acceptor (HBA), while urea, organic acids, sugars and polyols were selected as hydrogen bond donors (HBDs) to prepare binary DESs. The results are shown in Table 1. As shown in Table 1, different DESs exhibited distinct extraction efficiencies for rubusoside. Among them, DESs prepared with polyols as HBDs showed relatively higher extraction capacities, especially DES-10 and DES-9, with extraction yields of 6.68% and 6.67%, respectively, both of which were higher than those of the water extraction group (6.50%). Based on these results, ternary DESs were further constructed using ChCl, 1,3-BD and 1,2-PG. The results showed that DES-16 (ChCl:1,2-PG:1,3-BD) achieved the highest extraction yield of 7.11%, representing a 9.38% increase compared with water extraction. This may be attributed to the important role of hydrogen-bonding interactions between DESs and the target compound, as the number of hydrogen bonds in the extraction system may affect the binding affinity between DESs and rubusoside molecules. Therefore, DES-16 was selected for subsequent molar-ratio optimization and extraction experiments.

### 3.2. Interaction Mechanism Between DES-16 and Rubusoside

#### 3.2.1. Thermophysical Properties and FTIR Characterization of DES-16

**Figure 2:**
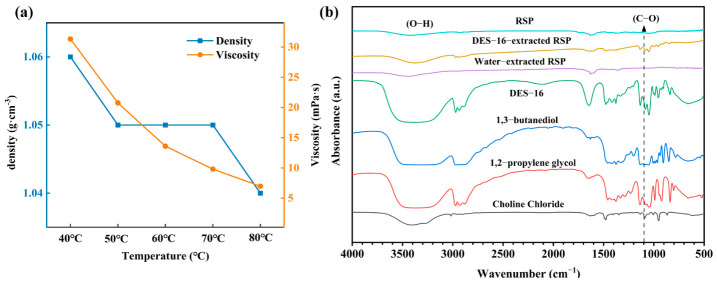
(**a**) Temperature-dependent density and viscosity of DES-16; (**b**) FTIR spectra of ChCl, 1,2-PG, 1,3-BD, DES-16, raw *R. chingii* var. *suavissimus* powder (RSP), water-extracted RSP and DES-16-extracted RSP.

Temperature is an important factor affecting the internal hydrogen-bonding network and fluid properties of DESs, and its variation directly influences the dissolution and mass transfer processes of target compounds [29]. Therefore, the density and viscosity of DES-16 were measured at different temperatures, and the results are shown in Figure 2a. As the temperature increased from 40 °C to 80 °C, both the density and viscosity of DES-16 decreased. Specifically, the density decreased from approximately 1.060 g/cm^3^ to 1.040 g/cm^3^, while the viscosity decreased markedly from 31.2 mPa·s to 6.5 mPa·s. The decrease in density was mainly attributed to thermal expansion of the DES system at elevated temperatures, which increased the intermolecular distance and thereby reduced the mass per unit volume. In contrast, viscosity was more sensitive to temperature changes. The sharp decrease in viscosity indicated that the internal hydrogen-bonding network of DESs was gradually weakened, molecular mobility was enhanced, and the fluidity of the system was significantly improved [30]. Lower viscosity is beneficial for improving solvent penetration into plant tissues and accelerating the mass transfer rate of target compounds [31], suggesting that increasing temperature contributes to improving the mass transfer performance of DESs and provides favorable conditions for the dissolution and release of rubusoside.

After clarifying the thermophysical behavior of DES-16, FTIR analysis was further performed to investigate its structural characteristics, and the results are shown in Figure 2b. Compared with the individual components, DES-16 retained the main characteristic absorption peaks of ChCl, 1,2-PG and 1,3-BD, while some peaks showed obvious shifts and broadening. In the region of 3200–3600 cm^−1^, DES-16 exhibited a broad and strong O–H stretching vibration band, with a broader peak shape than those of the individual components, indicating changes in hydroxyl-related interactions within the system [32]. In addition, an obvious C–O stretching vibration peak was observed at around 1050 cm^−1^, which appeared in both DES-16 and rubusoside samples. This reflected the presence of abundant C–O bonds in both systems, including oxygen-containing functional groups such as hydroxyl and glycosidic bonds [33], which may provide potential sites for subsequent intermolecular interactions. These results indicate that a new hydrogen-bonding network was formed between ChCl and the two polyols, confirming the successful construction of DES-16. Meanwhile, the hydroxyl and glycosidic bond structures in rubusoside may participate in molecular interactions with DESs.

To further verify whether interactions occurred between DESs and rubusoside, the FTIR spectra of the DES-16-extracted product and the water-extracted product were compared. The main characteristic peak positions of the two samples were generally consistent, with no new absorption peaks or disappearance of characteristic peaks observed. This indicated that microwave-assisted DES extraction did not alter the basic chemical structure of rubusoside, and that the enhancement effect of DESs during extraction was more likely derived from intermolecular interactions rather than chemical structural transformation [34]. In addition, compared with the water-extracted sample, the DES-16-extracted sample showed a slightly enhanced absorption band and a slight shift in the O–H stretching vibration region, suggesting the possible presence of hydrogen-bonding interactions between DESs and rubusoside. Previous studies have shown that the binding between DESs and glycoside-type natural products mainly depends on hydrogen-bonding networks formed by hydroxyl groups [35]. However, FTIR can only reflect the possible interactions between DESs and rubusoside from the perspective of functional group changes, while the specific interaction sites and interaction types still require further clarification. Therefore, considering the complex structure of rubusoside and the abundance of hydroxyl groups in its glucosyl moiety, glucose was selected as a representative model molecule in this study, and DFT calculations combined with ESP, AIM and IGMH analyses were further performed to reveal the intermolecular interaction mechanism between DES-16 and rubusoside.

#### 3.2.2. ESP Analysis

**Figure 3:**
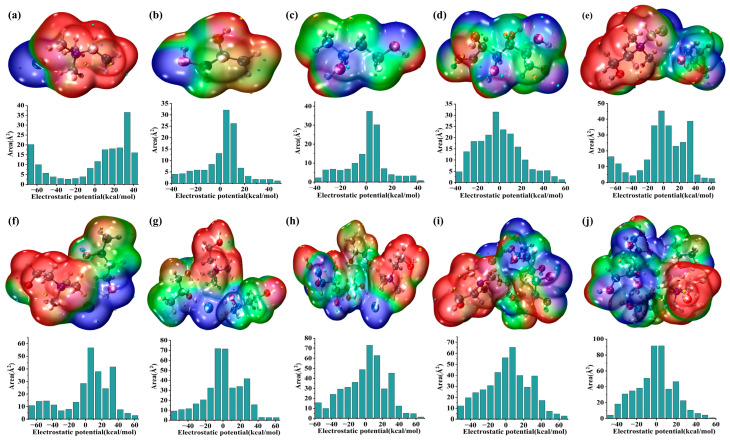
ESP and its quantitative distribution of (**a**) ChCl; (**b**) PG; (**c**) BD; (**d**) Glucose; (**e**) ChCl-PG; (**f**) ChCl-BD; (**g**) ChCl-BD-PG; (**h**) ChCl-PG-Glucose complex; (**i**) ChCl-BD-Glucose complex; (**j**) ChCl-BD-PG-Glucose complex.

To further elucidate the potential intermolecular interaction mechanism between DES-16 and rubusoside, electrostatic potential (ESP) analysis was performed on ChCl, 1,2-PG, 1,3-BD, glucose and their complexes, and the results are shown in Figure 3. In the ESP surfaces, the red regions represent positive electrostatic potential, whereas the blue regions represent negative electrostatic potential, corresponding to potential hydrogen-bond donor and hydrogen-bond acceptor regions, respectively. For the individual molecules, ChCl exhibited an obvious charge separation feature, with the positive electrostatic potential mainly distributed around the choline cation and the negative electrostatic potential concentrated near Cl^−^. PG (Figure 3b), BD (Figure 3c) and glucose (Figure 3d) showed similar ESP distribution patterns, in which the positive electrostatic potential was mainly located around hydroxyl hydrogen atoms, while the negative electrostatic potential was concentrated around oxygen atoms. These ESP distribution characteristics indicate that Cl^−^ has a strong hydrogen-bond acceptor ability, while the hydroxyl groups in PG, BD and glucose can act as both hydrogen-bond donors and acceptors, providing potential interaction sites for subsequent intermolecular interactions.

After ChCl formed binary systems with PG and BD, respectively (Figure 3e,f), more obvious complementary distributions of positive and negative electrostatic potential regions appeared at the molecular interfaces. When the ChCl-BD-PG ternary DES was further constructed (Figure 3g), the surface electrostatic potential distribution became more continuous and complex, while abundant positive and negative electrostatic potential regions were retained. Han [36] reported that the formation of DESs mainly depends on hydrogen-bonding interactions between HBAs and HBDs, and that the number and spatial distribution of hydroxyl groups in polyol-based HBDs can significantly affect the hydrogen-bonding network structure and solvation ability of DESs. With the increase in DES components, the number of potential interaction sites increased markedly, and the complementarity between positive and negative electrostatic potential regions was further enhanced, indicating that the system had greater potential to form richer intermolecular interactions. Previous studies have shown that electrostatic interactions and hydrogen-bonding interactions between DESs and target compounds are important driving forces for improving the solubility and extraction efficiency of natural products [37,38]. Therefore, compared with binary systems, the ternary DES was more favorable for establishing a multi-site cooperative interaction network with target molecules and had the potential to form a stable complex structure.

After the introduction of glucose, the ESP distributions of the ChCl-PG-glucose, ChCl-BD-glucose and ChCl-BD-PG-glucose complexes were further reconstructed (Figure 3h–j). Among them, the ChCl-BD-PG-glucose system exhibited the most obvious complementary distribution of positive and negative electrostatic potential regions, and multiple potential interaction sites were observed between the hydroxyl regions around glucose and Cl^−^ as well as the polyol oxygen atoms in DESs. Meanwhile, the quantitative ESP distribution showed a marked increase in the proportion of the moderate electrostatic potential region, suggesting that electron density redistribution occurred during complex formation [39]. Electron density redistribution is usually closely associated with the establishment of intermolecular interactions, indicating that obvious electron density rearrangement occurred between glucose and DESs, which was favorable for the formation of a stable complex structure. Previous studies have shown that multi-site hydrogen bonding and electron density redistribution can enhance the molecular affinity between DESs and glycoside compounds, thereby promoting the dissolution and release of target compounds [40]. Therefore, based on the ESP analysis in this study, it can be concluded that Cl^−^, PG and BD in DES-16 can form multiple potential interaction sites with glucose and establish a cooperative non-covalent interaction pattern. Considering that glucose is a representative structural unit of the glycosyl moiety of rubusoside, a similar interaction pattern may exist between DES-16 and rubusoside, thereby enhancing their molecular affinity and promoting the dissolution and release of rubusoside.

#### 3.2.3. AIM Analysis

**Figure 4:**
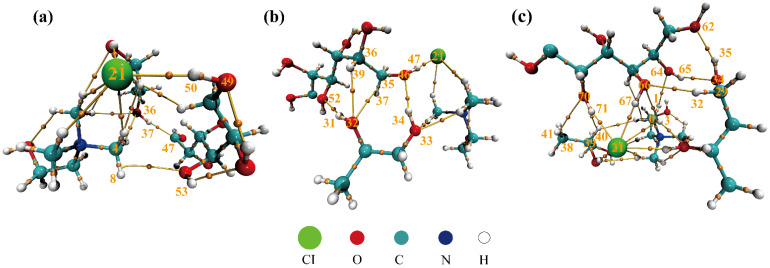
Bond Critical Point (BCP) plots of (**a**) ChCl-BD-glucose complex; (**b**) ChCl-PG-glucose complex; (**c**) ChCl-BD-PG-glucose complex. Note: The yellow lines denote bond paths of intermolecular interactions, and the orange spheres represent the corresponding bond critical points and orange numbers indicate the atom labels.

To further characterize the nature and strength of non-covalent interactions between DES and glucose, topological analysis was performed using atoms-in-molecules (AIM) theory. The ChCl-BD-glucose, ChCl-PG-glucose and ChCl-BD-PG-glucose complexes were analyzed, and the results are shown in Figure 4 and Table 2. In Figure 4, the yellow bond paths represent intermolecular interaction pathways, while the orange spheres indicate the corresponding bond critical points (BCPs). As observed in Figure 4, multiple intermolecular BCPs were formed in all three complex systems, indicating extensive intermolecular interactions between DES components and glucose. Specifically, the ChCl-BD-glucose and ChCl-PG-glucose systems formed 3 and 5 intermolecular BCPs, respectively, whereas the ChCl-BD-PG-glucose system formed 9 BCPs. According to AIM theory, the presence of BCPs is an important criterion for identifying intermolecular interactions, and the electron density ρ(r), Laplacian ∇^2^ρ(r) and potential energy density V(r) can reflect the nature and strength of these interactions [41]. Therefore, the greater number of BCPs in the ternary system indicates that it possesses more potential interaction sites and may form a richer intermolecular interaction pattern.

Table 2 further shows that the electron density *ρ*(*r*) of all BCPs ranged from 0.0041 to 0.0389 a.u., while the Laplacian ∇^2^*ρ*(*r*) values were all positive, ranging from 0.0168 to 0.0898 a.u., and the potential energy density *V*(*r*) values were negative. According to AIM criteria, positive ∇^2^*ρ*(*r*) together with negative *V*(*r*) generally corresponds to stable hydrogen-bonding interactions with closed-shell characteristics [42,43]. This indicates that the interactions formed between DES and glucose can provide stabilization contributions to the system. For the ChCl-BD-glucose system, O46-H47···Cl21 and O36-H37···C47 exhibited relatively high electron density and hydrogen-bond energy, with EHB values of 9.10 and 7.59 kcal/mol, respectively. For the ChCl-PG-glucose system, the hydrogen-bond energies of O33-H34···O46 and O32-H31···O52 reached 7.53 and 6.28 kcal/mol, respectively, indicating that Cl^−^ and hydroxyl oxygen atoms in polyols can act as important interaction sites involved in intermolecular binding. In contrast, the ChCl-BD-PG-glucose system not only formed more interactions, but also contained hydrogen bonds with different strength levels, including strong, moderate and weak interactions. Among them, the hydrogen-bond energies of O64-H65···O34 and O34-H35···O62 reached 7.37 and 6.68 kcal/mol, respectively, while those of O66-H67···Cl21 and O70-H71···Cl21 reached 5.74 and 5.62 kcal/mol, respectively. These results indicate that all components in the ternary DES can participate in hydrogen-bond construction and jointly form a multi-site cooperative interaction network.

It is worth noting that although the strength of individual hydrogen bonds in the ternary system was not the highest, this system showed the largest number of BCPs and a richer combination of hydrogen-bonding patterns. As shown in Table 2, the advantage of DES-16 did not arise from a single strong hydrogen bond, but rather from the synergistic effect of multiple moderate-strength hydrogen bonds. Previous studies have shown that, in polyhydroxy compounds and saccharide molecular systems, multi-site hydrogen-bonding networks are often more favorable than a single strong hydrogen bond for improving molecular binding stability and system adaptability [44]. Combined with the FTIR and ESP results, these findings indicate that Cl^−^, PG and BD in DES-16 can simultaneously establish multiple interactions with the hydroxyl groups of glucose, forming a synergistically stable hydrogen-bonding network. Since glucose is the major hydroxyl-rich structural unit of rubusoside, it can be inferred that a similar multi-site cooperative hydrogen-bonding interaction pattern may also exist between DES-16 and rubusoside, thereby enhancing their molecular recognition ability and binding stability and promoting the dissolution and release of rubusoside.

#### 3.2.4. IGMH Analysis

**Figure 5:**
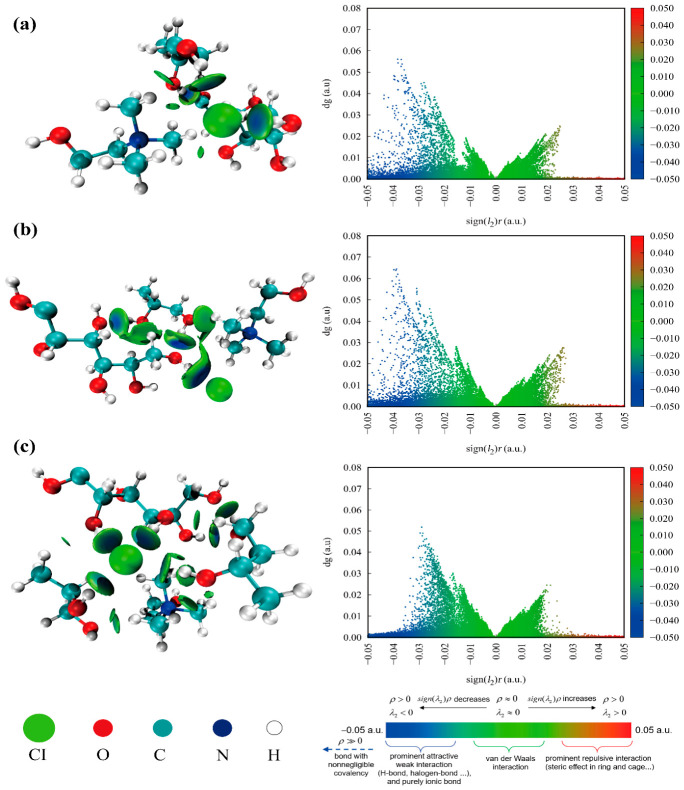
Elucidation of non-covalent interactions between glucose and ChCl-based DES via IGMH analysis: (**a**) ChCl-BD-glucose complex; (**b**) ChCl-PG-glucose complex; (**c**) ChCl-BD-PG-glucose complex.

To further visualize the spatial distribution characteristics of non-covalent interactions between DES and glucose, and to clarify the contribution of different interaction types to system stabilization, IGMH analysis was performed on the ChCl-BD-glucose, ChCl-PG-glucose and ChCl-BD-PG-glucose complexes, and the results are shown in Figure 5. The IGMH method can effectively distinguish different types of non-covalent interactions, and the isosurface color is mapped by *sign*(*λ*_2_)*ρ*, where blue regions represent strong attractive interactions such as hydrogen bonds, green regions mainly correspond to van der Waals interactions, and red regions indicate steric hindrance and repulsive interactions [45]. As shown in Figure 5a,b, obvious blue-green isosurfaces appeared in both the ChCl-BD-glucose and ChCl-PG-glucose systems, mainly distributed between the hydroxyl groups of glucose and Cl^−^, BD or PG hydroxyl groups. Meanwhile, in the corresponding *sign*(*λ*_2_)*ρ* scatter plots, a large number of points were concentrated in the negative region and mainly distributed in the low electron density region (*ρ* < 0.02 a.u.). Previous studies have shown that the appearance of blue-green isosurfaces usually indicates the coexistence of hydrogen bonding and dispersion interactions in the system, and these weak interactions make important contributions to the stabilization of complex systems [46]. In addition, when scatter points are mainly distributed in the negative *sign*(*λ*_2_)*ρ* region with relatively low electron density, it generally reflects the joint participation of hydrogen bonding and van der Waals interactions in system stabilization [47]. Therefore, non-covalent interactions involving both hydrogen bonding and van der Waals interactions existed between the binary DESs and glucose.

In contrast, the ChCl-BD-PG-glucose system (Figure 5c) exhibited larger and more continuous blue-green isosurfaces, which not only covered the region between glucose and Cl^−^, but also extended to the vicinity of BD and PG hydroxyl groups. According to the scatter plot, this system showed a wider distribution range and higher point density in the negative *sign*(*λ*_2_)*ρ* region. Previous studies have indicated that large green IGMH isosurfaces usually reflect extensive dispersion interactions and weak attractive forces between molecules, which can promote close molecular contact and enhance overall binding stability [48,49]. Meanwhile, the higher point density indicates the presence of more numerous and diverse attractive interactions in the system [50]. In addition, the peak of the scatter distribution was mainly concentrated in the medium-electron-density region, indicating obvious electron density redistribution and intermolecular charge reorganization during complex formation [51]. Such electron cloud rearrangement is usually accompanied by the formation of hydrogen-bonding networks and helps strengthen intermolecular electrostatic attraction and binding stability, thereby improving the molecular affinity and solvation ability of the system. Therefore, compared with the binary systems, the ternary DES formed a more extensive multi-site cooperative non-covalent interaction network with glucose.

Taken together, the ESP, AIM and IGMH results indicate that the binding between DES-16 and glucose did not depend on a single strong hydrogen bond, but was derived from a multi-site cooperative non-covalent interaction network jointly formed by multiple interaction sites in Cl^−^, PG and BD [52]. Specifically, AIM analysis confirmed the existence of multi-site hydrogen bonds and their stabilizing contributions, while IGMH results further revealed that, in addition to hydrogen bonding, widely distributed van der Waals interactions also participated in the stabilization of the system [53]. Since glucose represents the hydroxyl-rich glycosyl structure of rubusoside, it can be inferred that a similar multi-site cooperative hydrogen bonding–van der Waals interaction network may also be formed between DES-16 and rubusoside. This interaction pattern is not only favorable for enhancing the binding stability and molecular affinity between DES and the target molecule, but also promotes the release of rubusoside from the plant matrix and improves its dissolution and mass transfer efficiency in the DES system, thereby providing a molecular-level explanation for the enhanced mechanism by which DES-16 promotes the efficient extraction of rubusoside.

### 3.3. Optimization of the Extraction Process

#### 3.3.1. Single-Factor Experiment

**Figure 6:**
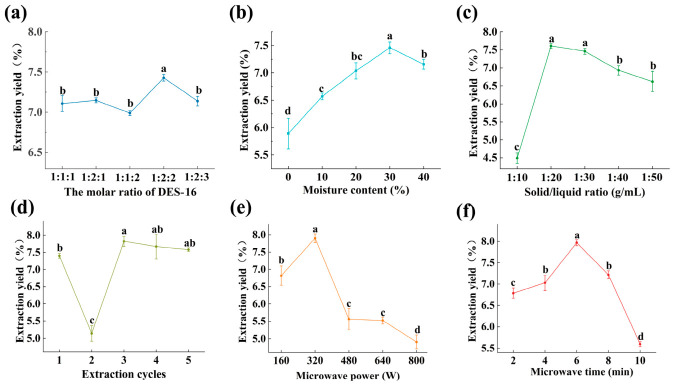
Effects of different single-factor conditions on rubusoside extraction yield during microwave-assisted extraction: (**a**) molar ratio of DES components; (**b**) moisture content; (**c**) solid-to-liquid ratio; (**d**) extraction cycles; (**e**) microwave power; and (**f**) microwave time. Different lowercase letters indicate significant differences among treatments within the same factor, as determined by one-way analysis of variance followed by Duncan’s multiple range test at *p* < 0.05.

After DES-16 was identified as the optimal extraction solvent, single-factor optimization was performed to further improve the extraction efficiency of rubusoside by investigating the main process parameters affecting the extraction process. The results are shown in Figure 6. As shown in Figure 6, different single-factor conditions significantly affected the extraction yield of rubusoside. With changes in the molar ratio of DES-16, the extraction yield first increased and then decreased, reaching the maximum value at ChCl:PG:BD = 1:2:2 (Figure 6a). As the moisture content increased, the extraction yield gradually increased and reached the highest value at 30%, followed by a decreasing trend (Figure 6b). When the solid-to-liquid ratio increased from 1:10 to 1:20 g/mL, the extraction yield increased markedly, whereas further increasing the solvent amount led to a decrease in extraction efficiency (Figure 6c). In addition, the number of extraction cycles also affected the recovery of rubusoside. The highest extraction yield was obtained when the extraction was performed three times, while further increasing the extraction number did not result in a significant improvement (Figure 6d). Microwave power and microwave time also showed a trend of initial promotion followed by inhibition. When the microwave power increased from 160 W to 320 W, the extraction yield increased significantly, whereas further increasing the power caused a decrease in yield (Figure 6e). Similarly, the extraction yield gradually increased with prolonged microwave time and reached the maximum at 6 min, followed by a decreasing trend (Figure 6f). These results indicate that each process parameter had an appropriate range. A suitable DES composition ratio was beneficial for constructing a stable hydrogen-bonding network and enhancing the dissolution capacity of DES toward rubusoside. An appropriate amount of water could reduce DES viscosity, improve system fluidity and promote mass transfer, whereas excessive water could weaken the internal hydrogen-bonding network of DES, thereby reducing extraction efficiency [54]. A moderate increase in the solid-to-liquid ratio could enhance the contact between solvent and raw material and promote the diffusion of the target compound. However, an excessively high solid-to-liquid ratio would reduce the microwave energy density absorbed per unit system, thereby weakening the microwave enhancement effect [55]. Meanwhile, appropriate microwave treatment could promote plant cell disruption and accelerate the migration of the target compound into the solvent, thereby improving extraction efficiency. However, excessive microwave power or prolonged treatment time may intensify local overheating, leading to the degradation or loss of rubusoside [56,57]. Moreover, when the extraction number exceeded three cycles, most extractable rubusoside in the raw material had already been released, and further increasing the extraction number did not substantially improve the recovery yield. Therefore, considering both extraction yield and process economy, the DES-16 molar ratio of 1:2:2, moisture content of 30%, solid-to-liquid ratio of 1:20 g/mL, extraction number of three cycles, microwave power of 320 W and microwave time of 6 min were selected as the center levels for the subsequent response surface optimization experiment.

#### 3.3.2. RSM for the Selection of Optimum Extraction Conditions

Based on the optimal levels identified in the single-factor experiments, the central levels and experimental ranges of moisture content (A), liquid–solid ratio (B), microwave time (C), and microwave power (D) were determined for subsequent response surface methodology (RSM) optimization of the microwave-assisted DES-16 extraction process. The BBD analysis of variance results and experimental results are shown in Table A1 and Table A2, respectively. The model was highly significant (*p* < 0.0001), while the lack-of-fit was not significant (*p* = 0.9735), and the *R*^2^ value was 0.9323, indicating that the model had good fitting and predictive ability. The established quadratic polynomial regression equation is shown in Equation (3). Although the interaction term AD was not statistically significant at the 0.05 level (*p* = 0.061), it was retained because the experimental data were fitted using a complete second-order polynomial model containing all linear, interaction, and quadratic terms. Therefore, AD was not interpreted as a significant interaction effect in the subsequent discussion.

Y = 7.89 + 0.4725 × A+0.2875 × B − 0.1058 × C − 0.1142 × D − 0.5125 × AB + 0.4000 × AC + 0.3450 × AD + 0.7500 × BC + 0.5550 × BD − 0.4625 × CD − 0.8532 × A^2^ − 0.8932 × B^2^ − 0.8683 × C^2^ − 0.7283 × D^2^ (3)

**Figure 7:**
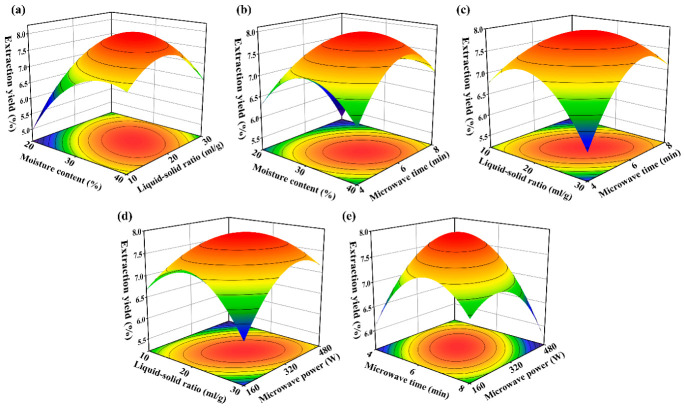
Three-dimensional response surface plots showing the effects of interactions among extraction variables on the rubusoside yield from *R. chingii* var. *suavissimus*: (**a**) moisture content and liquid–solid ratio; (**b**) moisture content and microwave time; (**c**) liquid–solid ratio and microwave time; (**d**) liquid–solid ratio and microwave power; and (**e**) microwave time and microwave power. Colors from blue/green to red indicate increasing predicted extraction yield.

The three-dimensional response surface plots and contour plots generated from the response surface model are shown in Figure 7. Overall, the response surfaces showed obvious convex characteristics, indicating a significant quadratic response relationship between extraction yield and the investigated factors. This was consistent with the results that A^2^, B^2^, C^2^ and D^2^ were all highly significant in the model (*p* < 0.001). Based on Table A1 and the regression equation (Equation (3)), the linear terms of moisture content (A) and liquid–solid ratio (B) significantly affected the extraction yield of rubusoside (*p* < 0.05), among which moisture content showed the most significant effect (*F* = 23.46). In addition, the interaction terms AB, AC, BC, BD and CD were all significant (*p* < 0.05), indicating obvious interactions among the investigated factors. Among them, BC (*F* = 45.33) and BD (*F* = 42.83) showed the most significant effects. The contour plots corresponding to BC and BD were clearly elliptical, and the response surfaces exhibited relatively large curvature, indicating strong interactions between the two factors. This further suggested that strong synergistic effects existed between liquid–solid ratio and microwave time, as well as between liquid–solid ratio and microwave power. These results indicated that the extraction efficiency of rubusoside was jointly regulated by the properties of the solvent system and microwave-assisted extraction conditions. An appropriate liquid–solid ratio could enhance the contact area between the solvent and raw material and promote the diffusion of the target compound, whereas an excessively high liquid–solid ratio might disperse microwave energy within a larger system volume, thereby reducing the energy density per unit system. Meanwhile, moderately increasing microwave power and extending microwave time were beneficial for promoting plant cell disruption and accelerating the release of the target compound. However, excessive microwave power or prolonged treatment time may intensify local overheating, leading to the degradation or loss of rubusoside [58]. Therefore, moisture content, liquid–solid ratio and microwave conditions showed obvious synergistic optimization relationships, and the proper matching of these factors was critical for achieving efficient extraction of rubusoside. Based on the established response surface model, numerical optimization of the process parameters was conducted to obtain the maximum extraction yield of rubusoside.

The results are shown in Table 3. The optimal conditions were determined as a moisture content of 33%, liquid–solid ratio of 21 mL/g, microwave time of 6 min and microwave power of 320 W, under which the predicted extraction yield was 7.97%. Verification experiments were then performed under these conditions, and the actual extraction yield reached 7.89 ± 0.25%, which was close to the predicted value, indicating that the model had good reliability and practical applicability. Moreover, compared with microwave-assisted water extraction conducted under identical conditions (6.50 ± 0.22%), the DES-16 system significantly improved the extraction efficiency of rubusoside, further confirming the superiority of DES-16 as an extraction solvent.

### 3.4. Morphological Characterization of R. chingii var. suavissimus Powder After Extraction

**Figure 8:**
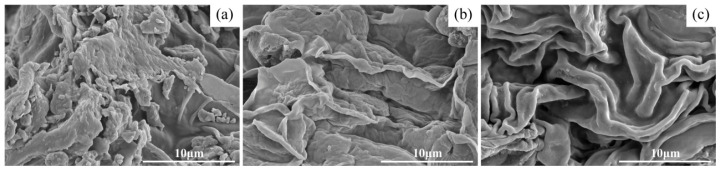
SEM images of *R. chingii* var. *suavissimus* powder after different extraction treatments: (**a**) raw *R. chingii* var. *suavissimus* powder; (**b**) residue after microwave-assisted water extraction; (**c**) residue after microwave-assisted DES-16 extraction. Scale bar = 10 μm.

To further reveal the effect of microwave-assisted DES extraction on plant tissue structure and verify its mass transfer enhancement mechanism, SEM observation was performed on raw *R. chingii* var. *suavissimus* powder and residues treated by different extraction methods, and the results are shown in Figure 8. The raw *R. chingii* var. *suavissimus* powder (Figure 8a) exhibited a relatively intact overall structure, with compact tissue arrangement, rough surfaces and obvious lamellar stacking, indicating that the plant cell structure was basically preserved. After microwave-assisted water extraction (Figure 8b), the sample surface showed a certain degree of wrinkling and loosening, and some lamellar structures were fractured, indicating that microwave treatment caused partial damage to the plant tissue. In contrast, the sample extracted by microwave-assisted DES-16 (Figure 8c) showed more obvious wrinkling, curling and loose structures, with extensive rupture of the original lamellar tissue. The degree of cell structure collapse was clearly greater than that of the water-extracted sample. Meanwhile, more cracks and pores appeared on the sample surface, indicating that the plant tissue underwent more severe structural disruption.

These pronounced microstructural changes suggested a synergistic effect between DES-16 and microwave treatment. Previous studies have shown that DESs can interact with cellulose, hemicellulose and lignin in plant cell walls through hydrogen bonding, thereby weakening the internal hydrogen-bonding network of the cell wall and reducing its structural stability [59,60]. Meanwhile, rapid microwave dielectric heating can promote intracellular water vaporization and pressure accumulation, thereby accelerating cell expansion and structural rupture [61]. Under the combined action of DES and microwave treatment, the integrity of plant tissues was further disrupted, and the cell wall and middle lamella structures gradually became loose, resulting in the formation of more cracks and pores. These results indicate that microwave-assisted DES-16 extraction was more effective than microwave-assisted water extraction in disrupting plant tissue structures, increasing cell wall permeability and shortening the mass transfer pathway, thereby promoting the diffusion and release of rubusoside from the plant matrix. This result is consistent with the improved extraction yield and the molecular interaction analysis described above, further confirming the synergistic extraction enhancement mechanism of DES and microwave treatment.

### 3.5. Rubusoside Separation and Sustainable Reuse of DES-16

**Figure 9:**
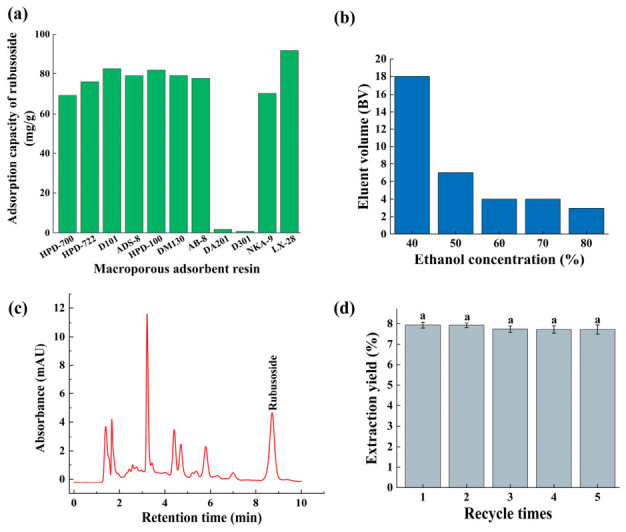
Rubusoside separation and DES recycling performance: (**a**) adsorption capacities of different macroporous resins; (**b**) elution volumes at different ethanol concentrations; (**c**) HPLC chromatogram of the rubusoside-enriched fraction after LX-28 resin purification; (**d**) extraction yields of the recovered DES during five reuse cycles. Lowercase letter indicate significant differences among recycling cycles, as determined by one-way analysis of variance followed by Duncan’s multiple range test at *p* < 0.05.

To achieve effective separation of rubusoside from DES-16 and evaluate the recyclability of DES, the adsorption capacity of different macroporous adsorption resins, elution conditions and DES reusability were investigated, and the results are shown in Figure 9. Significant differences were observed in the adsorption capacities of different macroporous adsorption resins toward rubusoside (Figure 9a). Among them, LX-28 exhibited the highest adsorption capacity, indicating its good selective adsorption ability for rubusoside. Therefore, LX-28 was selected for subsequent separation and purification. After the extract passed through the LX-28 resin column at a flow rate of 0.5 mL/min, rubusoside was adsorbed onto the resin, while DES-16 preferentially flowed out with the mobile phase, thereby achieving preliminary separation of the two components.

After LX-28 was identified as the optimal adsorption resin, the elution conditions were further optimized (Figure 9b). As the ethanol concentration increased from 40% to 80%, the elution volume of rubusoside gradually decreased. Notably, effective elution was achieved with only approximately 3 BV of 80% ethanol, indicating that a higher ethanol concentration was beneficial for improving the desorption efficiency of rubusoside. Therefore, 80% ethanol was finally used for elution at a flow rate of 0.5 mL/min to achieve efficient recovery of rubusoside. As shown in Figure 9c, the integrated peak area of rubusoside was 75.6, and the purity of the freeze-dried fraction was calculated to be 41.3% using the external-standard method.

The recyclability of DES is a key indicator for evaluating its industrial application potential and green sustainability. As shown in Figure 9d, after five consecutive reuse cycles, DES-16 maintained rubusoside extraction yields above 7.7%, with no significant difference among cycles (*p* > 0.05), indicating that DES-16 possessed good stability and reusability. The excellent recyclability of DES can effectively reduce the need for solvent replenishment and waste generation, making it an important approach to achieving green extraction and lowering production costs [62]. Meanwhile, good recovery performance is also an important indicator for evaluating the feasibility of industrial DES application. The recovered DES-16 after resin separation still maintained a high rubusoside extraction efficiency, suggesting that its key physicochemical properties remained relatively stable during repeated cycles. Therefore, DES-16 combines high extraction efficiency with good recyclability, demonstrating promising industrial application prospects and sustainable development potential.

## 4. Conclusions

In this study, a microwave-assisted deep eutectic solvent (DES) extraction system was developed to achieve the efficient and green extraction of rubusoside from Guangxi sweet tea (*R. chingii* var. *suavissimus*). The screening results showed that the ternary DES-16 system (ChCl:1,2-propylene glycol:1,3-butanediol = 1:2:2) exhibited the best extraction performance. Response surface optimization indicated that the optimal extraction conditions were a moisture content of 33%, liquid–solid ratio of 21 mL/g, microwave time of 6 min and microwave power of 320 W. Under these conditions, the rubusoside extraction yield reached 7.89 ± 0.25%, which was significantly higher than that obtained by microwave-assisted water extraction (6.50 ± 0.22%) and conventional hot water extraction (3.05 ± 0.25%).

FTIR, ESP, AIM, IGMH, and SEM analyses indicated that cooperative non-covalent interactions existed between DES-16 and rubusoside, and that DES-16 acted synergistically with microwave treatment to promote plant tissue disruption and mass transfer, thereby improving the dissolution and release efficiency of rubusoside. In addition, LX-28 resin enabled the recovery of a rubusoside-enriched fraction with a rubusoside purity of 41.3%, while the recovered DES maintained an extraction yield above 7.7% after five consecutive reuse cycles, demonstrating good stability and reusability. Therefore, microwave-assisted DES extraction represents an efficient, green, and recyclable strategy for rubusoside recovery. This study elucidated the cooperative interaction mechanism between DES and the glycosyl moiety of rubusoside and provides a useful reference for the extraction of natural glycoside-type bioactive compounds. However, given the moderate purity of the recovered rubusoside fraction, further optimization of the separation and purification process is required to improve product purity. Moreover, the toxicological safety of DES-16 and its residual levels in the final rubusoside-enriched fraction require further evaluation before practical application in food systems.

## Figures and Tables

**Table 1:** Effects of different DESs on rubusoside extraction yield from *R. chingii* var. *suavissimus*.

Abbreviation	HBA:HBD (Molar Ratio)	Extraction Yield (%)
DES-1	ChCl:urea (1:2)	6.32 ± 0.07
DES-2	ChCl:glucose (1:2)	6.48 ± 0.17
DES-3	ChCl:acetic acid (1:2)	5.04 ± 0.24
DES-4	ChCl:citric acid (1:2)	5.84 ± 0.36
DES-5	ChCl:D-sorbitol (1:2)	5.81 ± 0.96
DES-6	ChCl:xylitol (1:2)	6.57 ± 0.43
DES-7	ChCl:glycerol (1:2)	6.09 ± 0.07
DES-8	ChCl:ethylene glycol (1:2)	6.38 ± 0.13
DES-9	ChCl:1,2-propylene glycol (1:2)	6.67 ± 0.17
DES-10	ChCl:1,3-butanediol (1:2)	6.68 ± 0.16
DES-11	ChCl:levulinic acid (1:2)	3.94 ± 0.72
DES-12	ChCl:lactic acid (1:2)	4.24 ± 0.56
DES-13	ChCl:malic acid (1:2)	5.33 ± 0.34
DES-14	ChCl:diethylene glycol (1:2)	5.93 ± 0.55
DES-15	1,2-propylene glycol:1,3-butanediol:xylitol (1:1:1)	6.89 ± 0.65
DES-16	ChCl:1,2-propylene glycol:1,3-butanediol (1:1:1)	7.11 ± 0.20
MAE-Water		6.50 ± 0.22
Conventional hot water extraction		3.05 ± 0.25

**Table 2:** The topological parameters at the BCPs and the binding energy of Hydrogen bonds formed between DES-glucose complexes.

Structure	Hydrogen Bonds	*ρ*(*r*) (a.u.)	∇^2^*ρ*(*r*) (a.u.)	*V*(*r*) (a.u.)	*E_HB_* (kcal/mol)
ChCl-BD-glucose complex	O_51_-H_50_···CI_21_	0.0279	0.0603	−0.0189	5.93
	C_4_-H_8_···O_53_	0.0074	0.0279	−0.0047	1.47
	O_36_-H_37_···C_47_	0.0368	0.0590	−0.0242	7.59
ChCl-PG-glucose complex	O_46_-H_47_···CI_21_	0.0389	0.0669	−0.0290	9.10
	O_33_-H_34_···O_46_	0.0309	0.0898	−0.0240	7.53
	O_32_-H_31_···O_52_	0.0256	0.0768	−0.0200	6.28
	C_36_-H_39_···O_32_	0.0111	0.0420	−0.0077	2.40
	C_34_-H_37_···O_32_	0.0063	0.0245	−0.0036	1.14
ChCl-BD-PG- glucose complex	C_38_-H_41_···O_70_	0.0076	0.0274	−0.0046	1.44
	C_39_-H_40_···O_70_	0.0041	0.0168	−0.0024	0.74
	O_70_-H_71_···CI_21_	0.0253	0.0569	−0.0179	5.62
	O_66_-H_67_···CI_21_	0.0257	0.0576	−0.0183	5.74
	C_29_-H_32_···O_66_	0.0125	0.0407	−0.0085	2.68
	O_64_-H_65_···O_34_	0.0292	0.0813	−0.0235	7.37
	C_0_-H_1_···O_64_	0.0126	0.0422	−0.0084	2.64
	O_34_-H_35_···O_62_	0.0269	0.0764	−0.0213	6.68
	C_0_-H_3_···O_66_	0.0050	0.0196	−0.0027	0.86

**Table 3:** Predicted and experimental yields at optimized conditions for microwave-assisted extraction.

Optimal Extraction Conditions				Rubusoside Extraction Yield (%)		
Moisture Content (%)	Liquid-Solid Ratio (mL/g)	Microwave Time (min)	Microwave Power (W)	Predicted	Experimental	MAE–Water
33	21	6	320	7.97	7.89 ± 0.25	6.50 ± 0.22

## Data Availability

The original contributions presented in this study are included in the article. Further inquiries can be directed to the corresponding author.

## Abbreviations

The following abbreviations are used in this manuscript:

AIM	Atoms-in-Molecules
DES	Deep Eutectic Solvent
DFT	Density Functional Theory
ESP	Electrostatic Potential
FTIR	Fourier Transform Infrared Spectroscopy
HPLC	High Performance Liquid Chromatography
IGMH	Independent Gradient Model based on Hirshfeld partition
RSM	Response Surface Methodology
SEM	Scanning Electron Microscopy

## Appendix A

**Table A1:** Analysis of variance (ANOVA) for the fitted response surface model of rubusoside extraction yield by microwave-assisted DES extraction.

Source	Extraction Yield of Rubusoside	
	F-Value	*p*-Value
Model	13.77	0.000
A-Moisture content	23.46	0.000
B-Liquid-solid ratio	8.69	0.011
C-Microwave time	1.18	0.296
D-Microwave power	1.37	0.261
A^2^	9.20	0.000
B^2^	5.61	0.000
C^2^	4.17	0.000
D^2^	19.71	0.000
AB	10.79	0.009
AC	7.49	0.033
AD	39.44	0.061
BC	45.33	0.001
BD	42.83	0.005
CD	30.13	0.016
Lack-of-fit	0.23	0.974

**Table A2:** Box–Behnken design of experiments and experimental results.

Variable Levels				Extraction Yield (%)
A: Moisture Content (%)	B: Liquid-Solid Ratio (mL/g)	C: Microwave Time (min)	D: Microwave Power (W)	
20	20	8	320	5.27
20	20	4	320	6.04
20	20	6	480	5.50
20	20	6	160	6.29
20	30	6	320	6.39
20	10	6	320	5.06
30	20	6	320	8.54
30	20	6	320	7.26
30	20	6	320	7.58
30	20	6	320	7.88
30	20	6	320	8.21
30	30	6	480	6.98
30	10	6	480	5.02
30	30	6	160	6.37
30	10	6	160	6.63
30	30	8	320	6.84
30	10	8	320	4.77
30	30	4	320	6.00
30	10	4	320	6.93
30	20	8	480	5.81
30	20	4	480	6.74
30	20	8	160	6.82
30	20	4	160	5.90
40	20	8	320	7.10
40	20	4	320	6.27
40	20	6	480	7.07
40	20	6	160	6.48
40	30	6	320	6.29
40	10	6	320	7.01
